# Intersection of reality and fiction in art perception: pictorial space, body sway and mental imagery

**DOI:** 10.1007/s10339-015-0702-0

**Published:** 2015-08-02

**Authors:** Joanna Ganczarek, V. Ruggieri, D. Nardi, M. Olivetti Belardinelli

**Affiliations:** Pedagogical University of Cracow, ul. Podchorazych 2, 30-084 Cracow, Poland; “Sapienza” University of Rome, via dei Marsi 78, 00185 Rome, Italy; Department of Psychology, Eastern Illinois University, 600 Lincoln Ave, Charleston, IL 61920 USA

**Keywords:** Pictorial space, Depth perception, Eye movements, Body sway, Mental imagery

## Abstract

**Background:**

The thesis of embodied cognition claims that perception of the environment entails a complex set of multisensory processes which forms a basis for the agent’s potential and immediate actions. However, in the case of artworks, an agent becomes an observer and action turns into a reaction. This raises questions about the presence of embodied or situated cognition involved in art reception.

**Aims:**

The study aimed to assess the bodily correlates of perceiving fictional pictorial spaces in the absence of a possibility of an actual physical immersion or manipulation of represented forms.

**Method:**

The subjects were presented with paintings by Vermeer and De Hooch, whilst their body sway and eye movements were recorded. Moreover, test and questionnaires on mental imagery (MRT, VVIQ and OSIQ) were administered.

**Results:**

Three major results were obtained: (1) the degree of pictorial depth did not influence body sway; (2) fixations to distant elements in paintings (i.e. backgrounds) were accompanied by an increase in body sway; and (3) mental rotation test scores correlated positively with body sway.

**Conclusions:**

Our results suggest that in certain cases—despite the fictional character of art—observers’ reactions resemble reactions to real stimuli. It is proposed that these reactions are mediated by mental imagery (e.g. mental rotation) that contributes to the act of representing alternative to real artistic spaces.

## Introduction

Amongst several properties of visual targets, the viewing distance influences observer’s body posture in an important way, i.e. body sway increases with increasing viewing distance (e.g. Paulus et al. 1989).

In the field of experimental aesthetics, it has been proposed that also the illusion of distance created with the use of static pictorial depth cues might lead to an increase in body sway (Kapoula et al. 2011). In this study, subjects’ posture was destabilised when viewing paintings containing multiple depth cues as well as when fixating background areas of the paintings. However, results of other studies (Ganczarek et al. 2012, 2013; Emoto et al. 2006) suggest that pictorial depth does not directly increase observers’ body sway, but its effect might be mediated by frequent fixations to apparently distant elements in the paintings and by mental imagery allowing a mental representation of depicted spaces.

The present study aimed to reinvestigate this pictorial depth effect with the use of a greater number of stimuli differing in the degree of depth. We combined eye tracking and posturography in order to observe the relationship between fixations to backgrounds and postural reaction. Also, the question of possible mediating factors was posed.

It is well documented that many cognitive factors influence postural balance (e.g. Isableu et al. 1997; Maylor et al. 2001). With respect to art experience, individual differences in mental imagery (object or spatial imagery) constitute an important factor that affects artwork processing and consequentially might affect the relationship between perception of pictorial spaces and posture as well. Based on the description provided in the object-spatial imagery questionnaire (OSIQ) (Blajenkova et al. 2006), it can be expected that object visualisers, i.e. those scoring high on the object scale and on the vividness of visual imagery questionnaire (VVIQ), will employ a picturesque, holistic processing related to appearances of individual objects within this space. On the other hand, spatial visualisers, i.e. those scoring high on the spatial scale and in the mental rotation test (MRT), will operate a sequential, analytic processing of relations between represented objects and multiple spatial frames of reference within the painting and with respect to the observer.

Given that mental rotation involves not just analogical representation of forms but motor simulation as well (for a review see: Zacks 2008), it is possible that postural stability of spatial visualisers would be affected when performing spatial transformations. On the contrary, vivid and picturesque processing of object visualisers will influence less their posture as it is usually associated with a strong activation of the primary visual cortex (Olivetti Belardinelli et al. 2009) and not of the motor areas.

To summarise, the research hypotheses are:

1. Increasing pictorial depth induces increased postural sway;
2. Fixations on background areas correlate positively with body sway;
3. Spatial abilities/spatial imagery correlate positively with body sway; vividness/object imagery correlates negatively with body sway.

## Method

Twenty-nine undergraduate students participated in the study (*M* = 31.1, *SD* = 5.5) (14 M; 15 F). The participants had no prior history of higher degree art training. They all had normal visual acuity or corrected to normal and no known neurological diseases, vestibular, muscular and/or other abnormalities.

The participants stood in the standardised Romberg position on the pressure plate (PedHa 40 × 40 sensors) in front of a 22'' monitor placed frontally at the distance of approx. 70 cm. The monitor was connected to the eye tracker (RED500; SMI; sampling frequency 500 Hz). The eye movements and body sway were recorded during three blocks of images all following the same instruction (‘You have 30 s of free exploration of each image. During the interval of 30 s between the images a black screen with a central cross is presented. Before the next image is presented I will ask you to fixate on the cross in the centre of the screen. Do not lower your gaze or turn your head during the whole session’). All stimuli within the blocks were presented in a random fashion. The first block contained two baseline stimuli (central cross subtending resp. 1° and 2° of visual angle), whereas the second one included five simple images varying in the number of monocular depth cues.

In the third block, participants viewed nine seventeenth-century Dutch paintings by Johannes Vermeer and Pieter de Hooch divided into three groups differing in the degree of depth (3 close-range, e.g. ‘The Milkmaid’/Vermeer; 3 mid-range, e.g. ‘The Concert’/Vermeer and 3 long-range ‘The Bedroom’/de Hooch). All images were converted to greyscale. They were selected due to the almost photographic rendering of everyday indoor scenes with the use multiple monocular depth cues.

After the measurement, MRT (Vandenberg and Kuse 1978), VVIQ (Marks 1973 as adapted by Palmiero et al. 2011) and the Italian version of OSIQ (Vannucci et al. 2006) were administered.

### Posturographic parameters

All postural parameters were computed from raw data obtained from the Riabyla software. The movement of the centre of pressure in the lateral (*SD* X) and anterior–posterior (*SD* Y) directions and the surface of an ellipse containing 95 % of the closest centre of pressure positions (CoP)[mm2] were computed accordingly. The raw positions of centre of pressure were filtered by removing the outliers above 2 *z*-scores.

### Eye-tracking parameters

Eye movements were recorded with a remote eye tracker (RED 500, SensoMotoric Instruments), with a frequency of sampling of 500 Hz and an accuracy of 0.4 deg. The data obtained were analysed with BeGaze. In each image, a background area of interest (AOI) was defined. The background area related to the elements located in the back of the pictorial space, apparently most distant from the viewer. In the case of long-range paintings, the backgrounds contained so-called *doorkijkje* (see-through doorway), i.e. openings to another rooms or outside views. For the correlations between body sway and eye movements, net dwell time (NDT)[ms] of fixations on a given area of interest was used as parameter.

## Results

### Effect of pictorial depth on body sway

Due to the positively skewed distribution of postural parameters (Shapiro–Wilk: *p* < .05), all analyses performed on the postural data were nonparametric (including the correlations) with Bonferroni correction for multiple comparisons.

A Friedman test was run to assess the influence of different spatial information (mean baseline condition +3 groups of paintings) on body sway. The analysis revealed no significant effect of depth for any of the postural parameters (*SD* X *χ*^2^(3) = 0.4, *p* = .9; *SD* Y *χ*^2^(3) = 3, *p* = .4; CoP *χ*^2^(3) = 0.8, *p* = .8) (see Table 1).

**Table 1:** Descriptive statistics for postural data for 3 groups of paintings: ‘close-range’ (close), ‘mid-range’ (mid) and ‘long-range’ (long) and baseline condition

	*N*	Median	Range
Close_*SD* X	29	1.0034	3.44
Close_*SD* Y	29	.2398	3.23
Close_CoP	29	6.1131	260.66
Mid_*SD* X	29	.9639	3.21
Mid_*SD* Y	29	.2835	.92
Mid_CoP	29	4.5420	158.89
Long_*SD* X	29	.9202	5.98
Long_*SD* Y	29	.2704	1.38
Long_CoP	29	5.8954	188.30
Baseline_*SD* X	29	1.1538	2.34
Baseline_*SD* Y	29	.2725	1.70
Baseline_CoP	29	5.0607	148.98

### Fixations to the backgrounds and body sway

The positive correlations between postural parameters and NDT of fixations to background areas were found for the ‘long-range’ paintings (*SD* X *r*_s_ = .5, *p* = .005; *SD* Y *r*_s_ = .42, *p* = .02; CoP *r*_s_ = .46, *p* = .01) but not for the other groups of paintings (close-range: *SD* X *r*_s_ = −.05, *p* = .8; *SD* Y *r*_s_ = −.14, *p* = .5; CoP *r*_s_ = −.1, *p* = .6 and mid-range *SD* X *r*_s_ = *.25*, *p* = .2; *SD* Y *r*_s_ = .07, *p* = .7; CoP *r*_s_ = .17, *p* = .36).

### MRT/VVIQ/OSIQ and body sway

In general, MRT scores correlated positively with body sway when viewing paintings (*SD* X *r*_s_ = .378, *p* = .04; *SD* Y *r*_s_ = .425, *p* = .002; CoP *r*_s_ = .41, *p* = .02). When analysing the correlations at each single level of the dependent variable (i.e. ‘close’, ‘mid’ and ‘long-range’ paintings), the correlations were significant only for the ‘long-range’ group of paintings (*SD* X *r*_s_ = .493, *p* = .007; *SD* Y *r*_s_ = .504, *p* = .005; CoP *r*_s_ = .41, *p* = .03). The VVIQ and OSIQ ratings did not correlate significantly with the postural parameters (VVIQ: *SD* X *r*_s_ = −.1, *p* = .4; *SD* Y *r*_s_ = −.04, *p* = .8; CoP *r*_s_ = −.02, *p* = .9; OSIQ object: *SD* X *r*_s_ = .2, *p* = .2; *SD* Y *r*_s_ = .04, *p* = .8; CoP *r*_s_ = .1, *p* = .5; OSIQ spatial: *SD* X *r*_s_ = 0, *p* = .9; *SD* Y *r*_s_ = .09, *p* = .6; CoP *r*_s_ = −.04, *p* = .8).

## Discussion

No significant differences were found in viewers’ body sway comparing the three groups of paintings. This result confirms the data obtained in previous studies (Ganczarek et al. 2012, 2013; Emoto et al. 2006) and does not support the idea presented by Kapoula et al. (2011) that pictorial depth provokes postural destabilisation. A possible explanation of the different experimental outcomes lies in the nature of presented stimuli and in the hypothetical mechanism inducing body sway. The paintings used by Kapoula et al. (2011) had a confusing pictorial space containing both depth and movement cues, whereas the works by Vermeer and Pieter de Hooch offered a highly defined and static pictorial space. Therefore, it is possible that viewer’s posture might be affected by mental spatial transformations needed to disambiguate complicated and conflicting spatial layouts and by movement cues rather than depth cues.

This interpretation is supported by the positive correlations found between fixations to backgrounds and viewer’s body sway in the case of the ‘long-range’ paintings only. It is plausible that the background elements in these paintings such as open windows with external landscapes or corridors exert the postural destabilisation through the effect of blur (e.g. Straube et al. 1990). However, it is not a likely explanation because fixations to similarly unclear elements in background areas in other groups of paintings did not induce postural instability. Therefore, possibly not just the blurred appearance of background elements plays a role but also their location within the complex spatial arrangement of long-range paintings. In this case, the postural destabilisation associated with frequent fixations to the backgrounds might be treated as an indication of spatial processing of elaborate structures. This view is corroborated by the results regarding the mental rotation test discussed below.

The positive correlation between MRT scores and viewer’s body sway suggests that body sway might be mediated by individual differences in mental imagery. Interestingly, this effect is significant only when viewing artworks characterised by multiple levels of pictorial depth, intricate arrangement of spatial units and effects of enclosures and apertures towards outdoor scenes. Due to this particularity, it is possible that subjects having better abilities of mental spatial transformations might have processed these paintings differently from other subjects and as a consequence their posture was destabilised through a form of motor simulation (e.g. Zacks 2008). Obviously, this explanation requires further investigation and other methods that could ascertain that the individual styles of mental imagery interfere and shape the ongoing perception of art.

The present study has also some limitations. Firstly, the paintings used come from a specific historical period, and it is possible that if other types of artworks were applied (e.g. abstract art), the postural response might be remarkably different. Moreover, the mediating effect of mental imagery styles has been inferred from correlations between questionnaire scores and body sway. For future research, it would be advantageous to investigate body sway in conditions explicitly involving the two processing styles of object and spatial visualisers.
